# ^135^Cs activity and ^135^Cs/^137^Cs atom ratio in environmental samples before and after the Fukushima Daiichi Nuclear Power Plant accident

**DOI:** 10.1038/srep24119

**Published:** 2016-04-07

**Authors:** Guosheng Yang, Hirofumi Tazoe, Masatoshi Yamada

**Affiliations:** 1 Department of Radiation Chemistry, Institute of Radiation Emergency Medicine, Hirosaki University, 66-1 Hon-cho, Hirosaki, Aomori 036-8564, Japan; 2 Division of Nuclear Technology and Applications, Institute of High Energy Physics, Chinese Academy of Sciences; Beijing Engineering Research Center of Radiographic Techniques and Equipment, Beijing 100049, China

## Abstract

^135^Cs/^137^Cs is a potential tracer for radiocesium source identification. However, due to the challenge to measure ^135^Cs, there were no ^135^Cs data available for Japanese environmental samples before the Fukushima Daiichi Nuclear Power Plant (FDNPP) accident. It was only 3 years after the accident that limited ^135^Cs values could be measured in heavily contaminated environmental samples. In the present study, activities of ^134^Cs, ^135^Cs, and ^137^Cs, along with their ratios in 67 soil and plant samples heavily and lightly contaminated by the FDNPP accident were measured by combining γ spectrometry with ICP-MS/MS. The arithmetic means of the ^134^Cs/^137^Cs activity ratio (1.033 ± 0.006) and ^135^Cs/^137^Cs atom ratio (0.334 ± 0.005) (decay corrected to March 11, 2011), from old leaves of plants collected immediately after the FDNPP accident, were confirmed to represent the FDNPP derived radiocesium signature. Subsequently, for the first time, trace ^135^Cs amounts before the FDNPP accident were deduced according to the contribution of global and FDNPP accident-derived fallout. Apart from two soil samples with a tiny global fallout contribution, contributions of global fallout radiocesium in other soil samples were observed to be 0.338%–52.6%. The obtained ^135^Cs/^137^Cs database will be useful for its application as a geochemical tracer in the future.

The Fukushima Daiichi Nuclear Power Plant (FDNPP) accident in 2011 released tremendous amounts of radionuclides into the terrestrial environment, and the volatile radionuclides, especially radiocesium, have contributed to most of the radioactivities^1^,^2^,^3^,^4^,^5^. According to recent studies, the total atmospheric released amounts of ^134^Cs, ^135^Cs, ^136^Cs, and ^137^Cs from the FDNPP accident were estimated to be 11.8–18, 6.74 × 10^−5^, 2.2–2.6, and 13–36 PBq, respectively^1^,^6^,^7^,^8^. Since it is easier to obtain the activities of ^134^Cs (t_1/2_ = 2.06 y) and ^137^Cs (t_1/2_ = 30.1 y) by conventional γ spectrometry, most studies of radiocesium have focused on ^134^Cs and ^137^Cs for risk assessment, decontamination, and source identification^5^,^7^,^8^,^9^,^10^,^11^. ^135^Cs (t_1/2_ = 2.3 × 10^6^ y) is not currently considered to be radiologically significant, but it is a major contributor to the long-term radionuclide inventory. Furthermore, in various recent studies, the ^135^Cs/^137^Cs ratio has been emphasized heavily as a powerful geochemical tracer and time marker, including its application for identifying nuclear contamination sources^6^,^12^,^13^,^14^, indicating nuclear power plant operations^15^,^16^, dating nuclear fuel burn-up samples^17^, performing source term attribution of unknown industrial emission sources^18^, studying erosion^19^, dating sediment^19^,^20^,^21^, and modifying the model of anthropogenic radionuclide dispersion^22^. To widen the application of ^135^Cs/^137^Cs ratio, it is desirable to establish the ratio database because it not only provides information apart from the ^134^Cs/^137^Cs ratio, but it can also overcome the application limit of the ^134^Cs/^137^Cs ratio due to the short half-life of ^134^Cs (2.06 y).

For source term attribution, data were mainly from soil samples^11^,^23^,^24^ and newly grown plant samples collected after the FDNPP accident^5^,^11^,^24^,^25^. These samples have a potential contribution from the global fallout that is direct for soil samples and indirect via soil-to-plant transfer for newly grown plant samples. Therefore, these data would not reflect the specific FDNPP derived radiocesium signature accurately. For example, two recent studies reported relatively higher ^135^Cs/^137^Cs atom ratios for the standard reference materials released by the Japan Society for Analytical Chemistry (JSAC)^14^,^26^. These values were somewhat higher than the data (0.32–0.41) from heavily contaminated samples^6^,^25^,^27^,^28^. This indicated a rising proportion of the contribution from global fallout and other background sources in lightly contaminated samples due to the FDNPP accident, since the ^134^Cs activities (<360 Bq kg^−1^, decay corrected to March 11, 2011) were relatively low for these standard reference materials. Therefore, increasing the numbers of data for lightly contaminated samples is also highly desired for the analysis of low ^135^Cs activities, especially for obtaining a database of ^135^Cs/^137^Cs atom ratios to illustrate the influence of the FDNPP accident.

However, measurement of ^135^Cs encounters a great challenge, and there were no ^135^Cs data available in Japanese environmental samples before the FDNPP accident. It was only 3 years after the FDNPP accident that limited ^135^Cs values could be measured and therefore reported in heavily contaminated environmental samples^6^,^25^,^27^,^28^. The challenge to measure ^135^Cs/^137^Cs in lightly contaminated samples is due to the isobaric (^135^Ba and ^137^Ba) and polyatomic (^95^Mo^40^Ar^+^, ^97^Mo^40^Ar^+^, ^119^Sn^16^O^+^, and ^121^Sb^16^O^+^) interferences, along with the peak tailing effect from ^133^Cs during ICP-MS analysis. These will result in higher experimental uncertainties in the measured ^135^Cs/^137^Cs, which may be insufficient to discriminate different sources.

Recently, an almost interference-free and non-peak tailing spectrum has been achieved by combining ammonium molybdophosphate adsorption, cation-exchange chromatography, and triple-quadrupole inductively coupled plasma-mass spectrometry (ICP-MS/MS) analysis^26^. The low detection limits of 2.59 × 10^−5^ and 3.28 Bq kg^−1^ for ^135^Cs and ^137^Cs make it possible to carry out precise ^135^Cs/^137^Cs ratio analysis in lightly contaminated samples.

In the present study, soil and plant samples, both heavily and lightly contaminated by the fallout from the FDNPP accident, were collected immediately after the accident. Subsequently, conventional γ spectrometry was combined with the most advanced ICP-MS/MS available to measure the activities of ^134^Cs, ^135^Cs, and ^137^Cs for studying their distribution and risk assessment. Furthermore, the activity ratios of ^134^Cs/^137^Cs and the atom ratios of ^135^Cs/^137^Cs were also obtained for (1) illustrating the precise radiocesium signature due to the FDNPP accident from almost global fallout-free samples; and (2) gaining new knowledge on the contributions of global fallout and FDNPP accident derived fallout for the lightly contaminated samples. A preliminary database of ^135^Cs/^137^Cs atom ratios due to the FDNPP accident was built in order to widely apply the ^135^Cs/^137^Cs ratio as a new tracer of radiocesium in the future. Finally, the background values of ^135^Cs and ^135^Cs/^137^Cs before the FDNPP accident were presented for the first time.

## Results

The activities of ^134^Cs, ^135^Cs and ^137^Cs, the activity ratios of ^134^Cs/^137^Cs, and the atom ratios of ^135^Cs/^137^Cs are shown in Table S1. When decay-corrected to March 11, 2011, the ^134^Cs and ^137^Cs activities in the soil covered wide ranges, from 12.9 to 113 kBq kg^−1^ and from 14.2 to 110 kBq kg^−1^ (in dry weight), respectively. Similarly, the highest ^135^Cs activity (0.500 ± 0.014 Bq kg^−1^) was 2 orders of magnitude higher than that of the lowest available value (0.005 ± 0.001 Bq kg^−1^), except for three paddy soil samples below the detection limit. Among these three samples, one was a paddy field that was covered by a greenhouse plastic cover and it had the lowest ^134^Cs and ^137^Cs activities (12.9 ± 4.3 and 14.2 ± 3.8 Bq kg^−1^, respectively). The arithmetic means of the ^134^Cs/^137^Cs activity ratio (1.033 ± 0.006) and ^135^Cs/^137^Cs atom ratio (0.334 ± 0.005), from the old leaves of plants collected immediately after the FDNPP accident, were confirmed to represent the FDNPP derived radiocesium signature. Subsequently, trace amount of ^135^Cs with the highest activity of 0.0212 ± 0.0024 Bq kg^−1^ and ^135^Cs/^137^Cs atom ratios up to 4.02 before the FDNPP accident were deduced according to the contribution of the global fallout and the FDNPP accident-derived fallout.

## Discussion

Higher radiocesium activities were observed in the northwest direction from the FDNPP, in agreement with the observation that the radionuclides were mainly deposited northwest of the site in a strip approximately 40 km in length^1^,^4^,^29^,^30^,^31^. Radiocesium was not released simultaneously from the fuel in the reactors and the spent fuel pools (SFPs) of Units 1–3, and the SFP of Unit 4, but sequentially over the time-span of several days^32^. Therefore, heterogeneous deposition of ^134^Cs, ^135^Cs, and ^137^Cs occurred on the ground, which was consistent with the deposition of other radionuclides^33^. These heterogeneous distributions were also influenced by the initial deposition parameters, e.g., local wind direction and precipitation^1^, and the post-depositional redistribution, e.g., transportation of the soil particle-bound radiocesium by the surface runoff^34^. It can be concluded that certain ^134^Cs/^137^Cs and ^135^Cs/^137^Cs atom ratios, other than the distribution of radiocesium activities, may provide valuable information about sources of radiocesium, by comparing them with the signatures of Units 1–4.

In order to reduce the influence of global fallout on obtaining more accurate ^134^Cs/^137^Cs and ^135^Cs/^137^Cs signatures for the FDNPP accident, extremely heavily contaminated plant leaves with higher ^134^Cs (0.076–0.205 MBq kg^−1^) and ^137^Cs (0.073–0.199 MBq kg^−1^) were also selected for the ^135^Cs study. As shown in Table S1, relatively higher ^135^Cs concentrations, varying from 0.320 to 0.881 Bq kg^−1^-wet weight, were observed as expected.

Because of the easiness and importance of its measurement, the ^134^Cs/^137^Cs activity ratio was immediately applied as a distinguishing indicator between the Chernobyl accident and the FDNPP accident fallout^1^,^24^, and as an index for evaluating the contamination from each FDNPP reactor unit^11^,^25^. Compared with soil samples, it is more plausible to employ old leaves of plants collected immediately after the FDNPP accident to illustrate the radiocesium signature of FDNPP accident; since old leaves of plants have less deposition of global fallout than that of soil, and plant leaves generally have non-accumulating characteristics for Cs via soil-to-plant transfer^35^. The ^134^Cs/^137^Cs activity ratios for the plant leave samples covered a narrow range of 1.02–1.04. Moreover, the ^135^Cs/^137^Cs atom ratios for the plant leaves were also in a narrow range of 0.326–0.338, which was comparable with the range of values from leaf litter samples (0.333–0.341) collected in May 2011^6^,^27^. On the other hand, wider ranges of 0.907–1.05 and 0.315–0.419 were observed for the ^134^Cs/^137^Cs activity ratios and ^135^Cs/^137^Cs atom ratios in soil samples, respectively, as reported by the Ministry of Economy Trade and Industry (METI)^36^ and other authors^11^,^23^,^24^.

**Figure 1:**
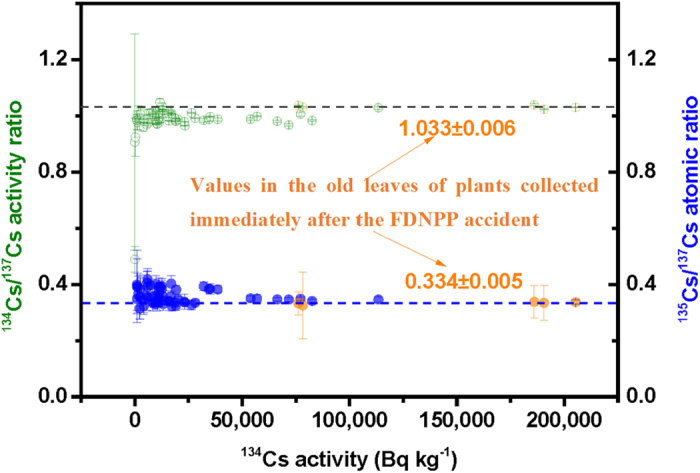
^134^Cs/^137^Cs activity ratios and ^135^Cs/^137^Cs atom ratios vs. ^134^Cs activities to illustrate the effects of both global and FDNPP accident fallout.

In terms of radionuclide signatures, radiocesium ratios released due to the Chernobyl accident, the FDNPP accident, and the global fallout from atmospheric nuclear weapon explosions were clearly distinct in Japan. Chernobyl-derived radiocesium had a lower ^134^Cs/^137^Cs activity ratio of 0.50–0.52 Bq/Bq (decay corrected to April 26, 1986)^37^, and presented a higher ^135^Cs/^137^Cs atom ratio of 0.480–0.589 (decay corrected to March 11, 2011)^6^,^12^,^13^,^18^,^26^,^27^,^38^. The global fallout radiocesium after the Chernobyl accident that fell in Japan had a ^134^Cs/^137^Cs activity ratio of 0.48–0.63 Bq/Bq (decay corrected to April 26, 1986)^39^. Unfortunately, the ^135^Cs/^137^Cs atom ratio in the global fallout of Japan is not available, and even values in other places were limited due to the challenge in measuring ^135^Cs^19^,^21^,^22^. Figure 1 presents the variations of ^134^Cs/^137^Cs activity ratios (in green) and ^135^Cs/^137^Cs atom ratios (in blue) compared with corresponding ^134^Cs activities in order to illustrate the effects of both global fallout and FDNPP accident fallout. Since the half-life of ^134^Cs is short (t_1/2_ = 2.06 y), the environmental ^134^Cs contamination before the FDNPP accident was basically zero. Therefore, the environmental ^134^Cs activities after the FDNPP accident indicated the contamination level due to this accident, that is, lower environmental ^134^Cs activities indicated a smaller proportion of radiocesium contamination from the FDNPP accident and a larger proportion of radiocesium contamination from the global fallout. The global fallout contribution to environmental samples would increase and it would become obvious in samples lightly contaminated by the FDNPP accident; that is, the ^134^Cs/^137^Cs activity ratio would present a decreasing trend and ^135^Cs/^137^Cs atom ratio would show an increasing trend for these samples. The reported higher ^135^Cs/^137^Cs atom ratios (0.3808–0.3896) from grass, bark, and moss, collected in August 2011, may have some influence from the soil-to-plant transfer of global fallout radiocesium 25. Sample S40 from a paddy field in Namie Town, with low ^134^Cs (84 ± 5 Bq kg^−1^; Table S1) and ^135^Cs below the detection limit, presented abnormally low ^134^Cs/^137^Cs activity ratios of 0.489 ± 0.045, indicating a larger contribution of ^137^Cs from global fallout for that collection site. Furthermore, the higher ^135^Cs/^137^Cs atom ratio of JSAC standard reference materials also indicated a larger contribution of global fallout and other background sources given their lower ^134^Cs activity (<360 Bq kg^−1^, decay corrected to March 11, 2011)^14^,^26^. In brief, radiocesium isotopic ratios, from the old leaves of plants collected immediately after the FDNPP accident, have great potential to represent the specific radiocesium signatures derived from the FDNPP accident.

For the source term identification, the evaluated isotopic compositions in the reactor cores of damaged Units 1–3 and in the SFPs have been frequently employed (Table 1). The results are given for irradiated uranium pellets and activated cladding tubes of zirconium alloy in the core and the SFPs of the respective reactors^40^. Radionuclides released into the stagnant water in the turbine buildings and their surroundings on the FDNPP site have been measured, providing a more accurate radioactive signature of the FDNPP accident^41^. The ^134^Cs/^137^Cs activity ratios and ^135^Cs/^137^Cs atom ratios in these two kinds of data were employed in the present study to illustrate radiocesium source for the FDNPP accident.

**Figure 2:**
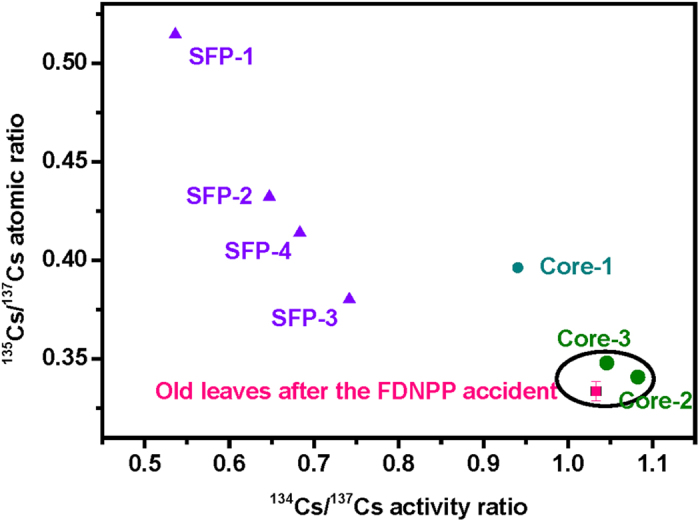
Comparisons of the ^134^Cs/^137^Cs activity ratios and ^135^Cs/^137^Cs atom ratios in the old leaves of plants collected immediately after the FDNPP accident (the present study), nuclear fuel in the damaged reactor cores, and nuclear fuel in the spent fuel pools (SFPs)^40^. The radiocesium isotopic compositions of the sampled leaves belonged to the cluster of Unit 2 and Unit 3.

**Figure 3:**
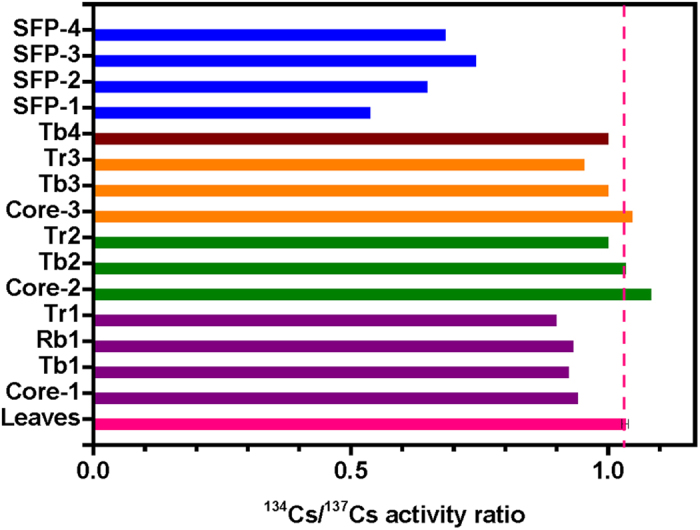
Comparisons the ^134^Cs/^137^Cs activity ratios in the old leaves of plants collected immediately after the FDNPP accident (the present study), nuclear fuel in the damaged reactor cores and in the spent fuel pools (SFPs)^40^, and stagnant water in the FDNPP (Tb, basement of the turbine building; Rb, basement of the reactor building; Tr, trench)^41^.

Figure 2 compares the ^134^Cs/^137^Cs activity ratios and ^135^Cs/^137^Cs atom ratios in the old leaves of plants collected immediately after the FDNPP accident, nuclear fuel in the damaged reactor cores, and nuclear fuel in the SFPs. The radiocesium isotopic compositions of all four SFPs were clearly different from those observed in the heavily contaminated leaves, indicating the potential release of radiocesium from SFPs was negligible, if any occurred. As shown in Table S2, the METI has estimated the released amounts of ^137^Cs from Unit 1, Unit 2, and Unit 3 were 3.86%, 91.5%, and 4.64%, respectively^36^. In a cluster analysis, the radiocesium isotopic compositions of sampled leaves belonged to the cluster of Unit 2 and Unit 3 (Figure 2). Because the ^135^Cs/^137^Cs atom ratios were not available in the stagnant water samples from the FDNPP, only ^134^Cs/^137^Cs activity ratios in the highly contaminated leaves were compared to that from the stagnant water in the basement of the turbine building (Tb), basement of the reactor building (Rb), and a trench (Tr), as shown in Figure 3. It was observed clearly that the ^134^Cs/^137^Cs activity ratio in the basement of Tb of Unit 2 was the closest value to that of the leaves.

All these findings further illustrated that the arithmetic means of the ^134^Cs/^137^Cs activity ratio (1.033 ± 0.006) and ^135^Cs/^137^Cs atom ratio (0.334 ± 0.005), from the old leaves of plants collected immediately after the FDNPP accident, could be applied as the specific FDNPP accident-derived radiocesium signatures to study the effect of the FDNPP accident on other lightly contaminated environmental samples.

Obtaining background data in the environment on radiocesium isotopic ratios is essential to widen their application as a geochemical tracer in the future and for assessment of the environmental impact of Cs released from the FDNPP accident. However, to the best of authors’ knowledge, there were no reported ^135^Cs/^137^Cs atom ratios in environmental samples before the FDNPP accident in Japan, and the first sets of ^135^Cs/^137^Cs atomic ratios in plants and soil^6^,^25^,^27^ and in rainwater^28^ were not reported until 3 years after the FDNPP accident, due to the challenge in measuring ^135^Cs. Before the FDNPP accident, the most recent major radiocesium contribution was from the Chernobyl accident. After the Chernobyl accident, the Meteorological Research Institute at Tsukuba City monitored 16 radionuclides, including ^134^Cs and ^137^Cs, in the fallout deposited in Japan during a monthly sampling program of the total fallout at eleven stations to obtain the temporal and spatial distributions of the radionuclides^39^. In the present study, the ^135^Cs background in the Fukushima area was obtained based on the following assumptions:

1. The radiocesium in Japanese environmental samples were from global fallout due to the combination of atmospheric nuclear weapon explosions and the Chernobyl accident, and fallout from the FDNPP accident;
2. The mean ^134^Cs/^137^Cs activity ratio of (2.13 ± 0.17) × 10^−4^ (Table S3) (decay corrected to March 11, 2011) in the wet and dry precipitation depositions from the eleven Japanese stations in 1986 was employed as the ^134^Cs/^137^Cs global fallout background activity ratio before the FDNPP accident;
3. The arithmetic means of ^134^Cs/^137^Cs activity ratio (1.033 ± 0.006) and ^135^Cs/^137^Cs atom ratio (0.334 ± 0.005), from the old leaves of plants collected immediately after the FDNPP accident, were employed as the specific FDNPP accident-derived radiocesium signature.

Subsequently, a simple two-end member mixing model was employed to calculate the relative contributions of the background radiocesium:


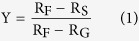


where R is the ^134^Cs/^137^Cs activity ratio or ^135^Cs/^137^Cs atom ratio; and subscripts F, G, and S refer to the FDNPP accident fallout, global fallout, and the soil sample, respectively. These relative contributions of the background radiocesium are shown in Table S4.

**Figure 4:**
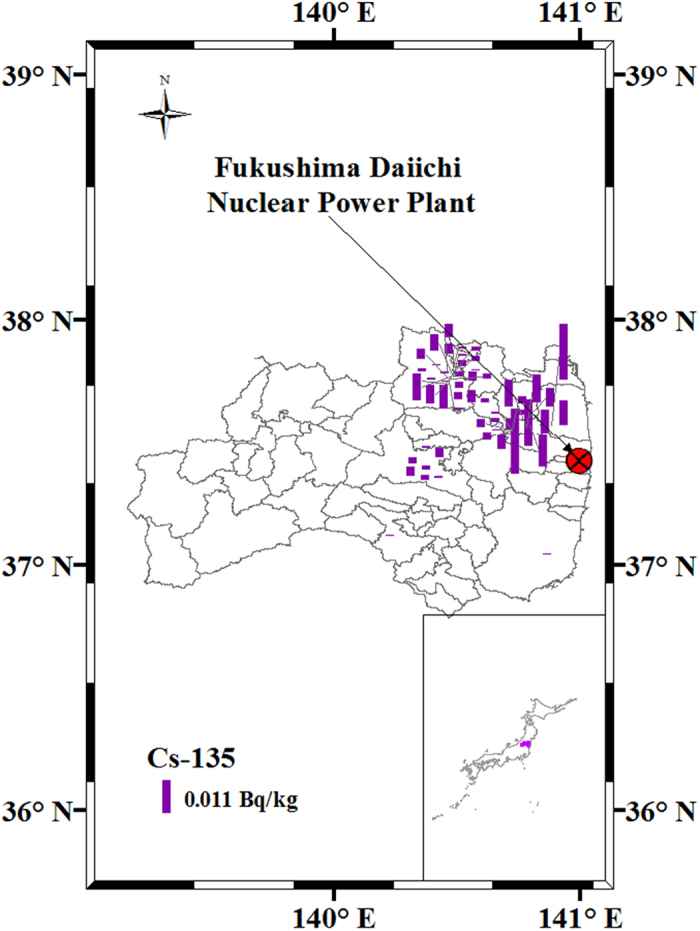
^135^Cs distribution in soil before the FDNPP accident. This map was prepared with Arc GIS 10.3 software (http://resources.arcgis.com/en/home/).

Apart from two soil samples with a tiny contribution from global fallout, the observed contribution of global fallout radiocesium ranged from 0.338% to 52.6% in all other samples. Sample S40 from a paddy field in Namie Town, with low ^134^Cs (84 ± 5 Bq kg^−1^; Table S1), had the highest contribution of global fallout radiocesium and it was abnormally high. The ^135^Cs background before the FDNPP accident was a trace amount, with data of five samples below the limit of detection. For other samples, a larger variation was observed, with the maximum value (0.0212 ± 0.0024 Bq kg^−1^) two orders of magnitude higher than the lowest value. As shown in Fig. 4, the soil samples collected close to the FDNPP had relatively higher ^135^Cs concentrations, which may have been due to the operation of the FDNPP from 1971 to 2011. Regarding the 82.1% availability of the ^135^Cs/^137^Cs atom ratios in the global fallout before the FDNPP accident, the range of 0.028–4.02 (decay corrected to March 11, 2011) was observed in the present study. These are the first batch of deduced values of ^135^Cs contents and ^135^Cs/^137^Cs atom ratios in global fallout before the FDNPP accident in Japan, and they can be employed in the future to illustrate the exact contribution of the FDNPP accident to the environment. It should be noted that more factors, such as the operation of the FDNPP, should be considered in the model to get a more accurate ^135^Cs background value in the future.

## Methods

### Soil and Plant Sampling

**Figure 5:**
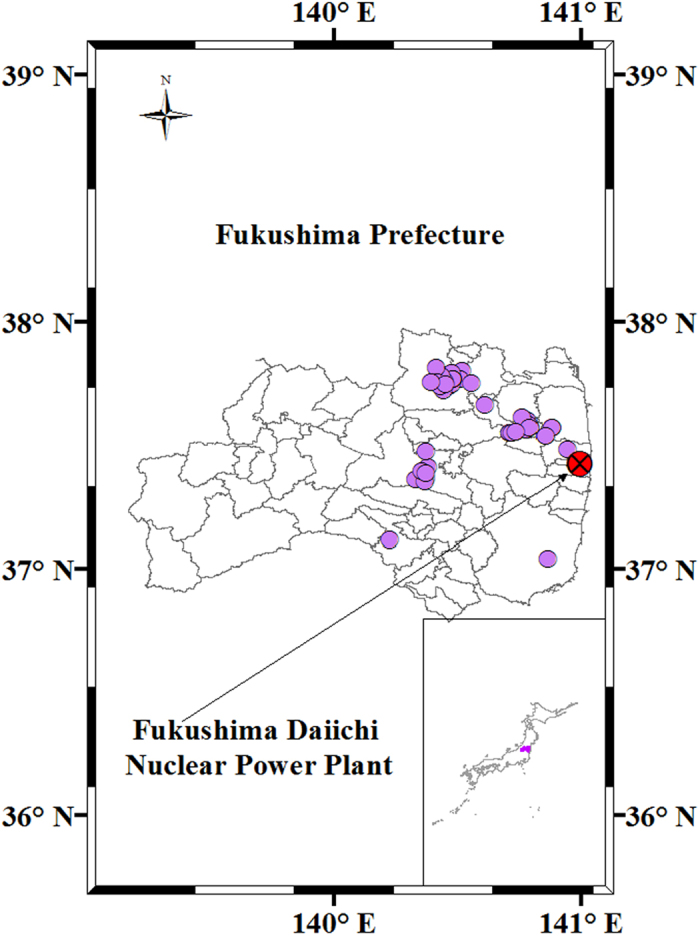
Sampling map; sampling sites are roughly marked with purple circles. This map was prepared with Arc GIS 10.3 software (http://resources.arcgis.com/en/home/).

The details of procedures for sampling and pretreating soil and plant samples have been described elsewhere^33^. Surface soils (0–5 cm) and plant leaves were collected from 67 sites in Fukushima Prefecture (Fig. 5) on four sampling expeditions in 2011, from April 12 to 16, April 26 to 28, June 6 to 10, and June 15 to 16. For the soil analysis, stones and plant roots were removed by handpicking and soil was transferred into a 100-mL polystyrene container. Leaves were collected from the upper part of the plant, so as not to be newly grown after the radionuclide contamination, to minimize soil-to-plant transfer from the contaminated soil. Then, they were cut into 1 × 1 cm pieces with scissors prior to further treatment.

### Measurement of Isotopes

The concentrations of ^134^Cs and ^137^Cs were determined by γ-ray spectroscopy (ORTEC GEM-40190, Seiko-EG&G, Tokyo, Japan) at energies of 604 keV and 662 keV, respectively. Mixed gamma standard sources distributed from the Japan Radioisotope Association were employed for efficiency correction. After organic matter decomposition in a muffle oven at 450 °C for 2 h, leaching with 20 mL of concentrated HNO_3_ was performed in PFA jars with lids (Savillex, Eden Prairie, MN, USA) on a hot plate at 180 °C for 2 h. During acid leaching, for those samples with high organic contents, 5 mL H_2_O_2_ was added. After filtration, the sample solutions were adjusted to 4 M HNO_3_ for Cs separation. The purification of Cs was conducted as described in Figure S1 following the method of Yang *et al*.^26^. Agilent 8800 (ICP-MS/MS, Agilent Technologies, Santa Clara, CA, USA) featuring an octopole collision/reaction cell situated between quadrupole mass filters (first, Q1; second, Q2) was employed for analysis of ^134^Cs/^137^Cs and ^135^Cs/^137^Cs ratios. The optimized operation parameters are summarized in Table S5. Finally, ^135^Cs activity could be obtained by combining the data from γ spectrometry and ICP-MS/MS.

## Additional Information

**How to cite this article**: Yang, G. *et al*.^135^Cs activity and ^135^Cs/^137^Cs atom ratio in environmental samples before and after the Fukushima Daiichi Nuclear Power Plant accident. *Sci. Rep.* **6**, 24119; doi: 10.1038/srep24119 (2016).

## Supplementary Material

Supplementary Information

## Figures and Tables

**Table 1:** Model calculation results of radiocesium isotopic compositions in Units 1–3 cores and fuel in the spent fuel pools (SFPs) obtained by the ORIGEN2 code, and the experimental ^134^Cs/^137^Cs activities ratios in the stagnant water in the FDNPP at the Tb, Rb and Tr sampling sites.

	Unit 1			Unit 2		Unit 3		Unit 4
Sampling site	Tb1	Rb1	Tr1	Tb2	Tr2	Tb3	Tr3	Tb4
Sampling date (2011)	3/26	5/27	3/29	3/27	3/30	4/22	3/30	4/21
^134^Cs/^137^Cs^a^	0.923	0.931	0.899	1.033	1.000	1.000	0.952	1.000
^136^Cs/^137^Cs^a^	0.185	0.259	0.165	0.247	0.245	0.256	0.257	0.259
	Core 1	SFP-1		Core 2	SFP-2	Core 3	SFP-3	SFP-4
^134^Cs/^137^Cs^b^	0.941	0.536		1.082	0.647	1.046	0.742	0.683
^135^Cs/^137^Cs^c^	0.396	0.515		0.341	0.432	0.348	0.380	0.414
^136^Cs/^137^Cs^b^	0.268	1.04 × 10^−9^		0.320	7.61 × 10^−6^	0.339	1.14 × 10^−7^	7.34 × 10^−4^

Tb: basement of the turbine building; Rb: basement of the reactor building; Tr: trench.

^134^Cs and ^137^Cs activities were decay corrected to March 11, 2011.

^a^Activity ratio reported by Nishihara *et al*.^40^.

^b^Activity ratio reported by Nishihara *et al*.^41^.

^c^Atom ratio reported by Nishihara *et al*.^41^.
