# Supplementary material for: 135Cs activity and 135Cs/137Cs atom ratio in environmental samples before and after the Fukushima Daiichi Nuclear Power Plant accident
Source: Sci Rep. 2016 Apr 7;6:24119. doi: 10.1038/srep24119 (PMC4823706; doi:10.1038/srep24119)
Supplement: Supplementary Information [file srep24119-s1.pdf]

Supporting Information

^{135}Cs activity and $^{135}Cs/^{137}Cs$ atom ratio in environmental samples before and after the Fukushima Daiichi Nuclear Power Plant accident

Guosheng Yang[1,2], Hirofumi Tazoe[1], Masatoshi Yamada[1,] *

[1] Department of Radiation Chemistry,

Institute of Radiation Emergency Medicine,

Hirosaki University,

66-1 Hon-cho, Hirosaki, Aomori 036-8564, Japan

[2]Division of Nuclear Technology and Applications, Institute of High Energy Physics,

Chinese Academy of Sciences;

Beijing Engineering Research Center of Radiographic Techniques and Equipment,

Beijing 100049, China

*Corresponding author. Tel.: +81 172 39 5405; Fax: +81 172 39 5405.

myamada@hirosaki-u.ac.jp

Table S1 | Cs radioisotopes in soil and plant samples contaminated by the FDNPP accident

**ID**	**Site**	**Collection date**	**Latitude (°N)**	**Longitude (°E)**	**^{134}Cs (Bq kg^{-1})**	**^{137}Cs (Bq kg^{-1})**	**^{135}Cs (Bq kg^{-1})**	**^{134}Cs/^{137}Cs activity ratio**	**^{135}Cs/^{137}Cs atom ratio**
S12	Minamisoma city	4/12/2011	37.5744	140.8822	82553±209	83909±223	0.376±0.007	0.984±0.004	0.341±0.006
S13	Minamisomac ity	4/12/2011	37.5744	140.8822	32065±133	32563±142	0.168±0.007	0.985±0.006	0.393±0.016
S14	Namie town	4/12/2011	37.5419	140.8594	34531±156	34945±167	0.176±0.003	0.988±0.006	0.383±0.006
S15	Namie town	4/13/2011	37.5733	140.7931	53877±178	54479±189	0.250±0.007	0.989±0.005	0.351±0.010
S16	Iitate village	4/13/2011	37.6036	140.7844	35024±23	35112±24	0.177±0.009	0.997±0.001	0.384±0.019
S19	Namie town	4/13/2011	37.5538	140.7216	5940±67	5897±67	0.030±0.004	1.007±0.016	0.393±0.054
S20	Namie town	4/13/2011	37.5538	140.7216	11270±86	11405±87	0.057±0.003	0.988±0.011	0.380±0.023
S23	Namie town	4/13/2011	37.5538	140.7216	11416±91	11485±91	0.059±0.005	0.994±0.011	0.393±0.030
S27	Namie town	4/13/2011	37.5538	140.7216	9870±81	10051±81	0.053±0.004	0.982±0.011	0.402±0.030
S29	Namie town	4/13/2011	37.5538	140.7216	5718±63	5782±63	0.031±0.003	0.989±0.015	0.406±0.041
S35	Namie town	4/14/2011	37.5538	140.7216	13±4	14±4	ND	0.907±0.385	ND
S36	Namie town	4/14/2011	37.5538	140.7216	288±15	311±16	ND	0.924±0.068	ND
S37	Namie town	4/14/2011	37.5538	140.7216	1537±33	1529±33	0.007±0.001	1.005±0.030	0.365±0.065
S39	Namie town	4/14/2011	37.5561	140.7398	12564±91	12146±90	0.061±0.006	1.034±0.011	0.384±0.037
S40	Namie town	2011/4/14	37.5519	140.7159	84±5	171±12	ND	0.489±0.045	ND
S42	Namie town	4/14/2011	37.5519	140.7159	16889±116	17042±117	0.090±0.007	0.991±0.010	0.403±0.029
S44	Namie town	4/14/2011	37.5519	140.7159	11698±95	11151±93	0.057±0.003	1.049±0.012	0.393±0.017
S45	Namie town	4/15/2011	37.5561	140.7398	19276±123	19622±124	0.098±0.004	0.982±0.009	0.381±0.014
S46	Namie town	4/15/2011	37.5651	140.7839	71703±200	74086±212	0.336±0.007	0.968±0.004	0.346±0.007
S47	Namie town	4/15/2011	37.5651	140.7839	38630±158	39096±165	0.196±0.007	0.988±0.006	0.383±0.013
S48	Namie town	4/15/2011	37.5651	140.7839	77179±481	76651±251	0.351±0.006	1.007±0.007	0.349±0.006
S49	Namie town	4/15/2011	37.5881	140.7919	66285±201	67534±210	0.308±0.005	0.982±0.004	0.347±0.006

S54	Iitate village	4/16/2011	37.6161	140.7672	113438±367	110208±375	0.500±0.014	1.029±0.005	0.346±0.010
S55	Shirakawa city	4/26/2011	37.1221	140.2285	2587±38	2569±38	0.013±0.001	1.007±0.021	0.384±0.040
S56	Iwaki city	4/26/2011	37.0451	140.8649	1313±27	1344±27	0.007±0.001	0.977±0.028	0.390±0.049
S57	Koriyama city	4/27/2011	37.3706	140.3752	7269±64	7188±64	0.037±0.002	1.011±0.013	0.398±0.022
S58	Fukushima city	4/27/2011	37.7628	140.4685	5866±58	5927±58	0.033±0.003	0.990±0.014	0.419±0.037
S59	Fukushima city	4/27/2011	37.7628	140.4684	12558±114	12479±80	0.065±0.003	1.006±0.011	0.395±0.021
S60	Aizuwakamatsu city	4/27/2011	37.4847	140.9451	982±24	997±24	0.005±0.001	0.985±0.034	0.348±0.053
S61	Kawamata town	4/28/2011	37.6686	140.6148	3981±46	4142±46	0.019±0.002	0.961±0.015	0.343±0.043
S62	Fukushima city	6/6/2011	37.7892	140.4583	20274±34	20529±34	0.093±0.003	0.988±0.002	0.347±0.011
S63	Fukushima city	6/6/2011	37.7741	140.4710	56913±74	56974±75	0.261±0.010	0.999±0.002	0.350±0.013
S64	Fukushima city	6/6/2011	37.7423	140.4676	981±4	988±4	0.005±0.002	0.992±0.006	0.393±0.129
S65	Fukushima city	6/6/2011	37.7508	140.4679	2388±12	2429±12	0.011±0.003	0.983±0.007	0.355±0.079
S66	Fukushima city	6/7/2011	37.7637	140.4686	15843±15	16111±16	0.072±0.003	0.983±0.001	0.340±0.012
S67	Fukushima city	6/7/2011	37.7656	140.4694	28071±24	28279±25	0.124±0.002	0.993±0.001	0.335±0.006
S68	Date city	6/8/2011	37.8052	140.5221	9717±17	9831±17	0.043±0.002	0.988±0.002	0.330±0.018
S69	Fukushima city	6/8/2011	37.7708	140.5144	19070±17	19425±17	0.083±0.004	0.982±0.001	0.325±0.016
S70	Fukushima city	6/8/2011	37.7563	140.5579	10305±15	10612±15	0.048±0.005	0.971±0.002	0.342±0.037
S71	Fukushima city	6/8/2011	37.7501	140.4782	23265±18	24089±18	0.106±0.003	0.966±0.001	0.337±0.008
S72	Fukushima city	6/9/2011	37.7246	140.4467	5518±12	5691±13	0.027±0.004	0.970±0.003	0.359±0.051
S73	Fukushima city	6/9/2011	37.7957	140.4789	12918±14	12628±14	0.059±0.003	1.023±0.002	0.359±0.021
S74	Fukushima city	6/9/2011	37.7719	140.4857	23039±29	23521±29	0.104±0.008	0.980±0.002	0.339±0.026
S75	Fukushima city	6/9/2011	37.7719	140.4857	8375±16	8480±16	0.040±0.003	0.988±0.003	0.357±0.027
S76	Fukushima city	6/9/2011	37.7719	140.4857	13173±22	12927±22	0.056±0.004	1.019±0.002	0.331±0.023
S77	Fukushima city	6/9/2011	37.7719	140.4857	26396±34	26107±34	0.112±0.006	1.011±0.002	0.327±0.018
S78	Fukushima city	6/9/2011	37.7719	140.4857	2198±9	2208±8	0.009±0.001	0.996±0.005	0.315±0.040
S79	Fukushima city	6/9/2011	37.7719	140.4857	18752±23	18914±23	0.083±0.003	0.991±0.002	0.336±0.013
S80	Fukushima city	6/9/2011	37.7719	140.4857	15885±26	15738±25	0.070±0.002	1.009±0.002	0.341±0.012
S81	Fukushima city	6/9/2011	37.7643	140.4483	11108±19	11179±19	0.049±0.002	0.994±0.002	0.335±0.013

S82	Fukushima city	6/9/2011	37.7849	140.4368	13673±20	13404±20	0.060±0.002	1.020±0.002	0.343±0.011
S83	Fukushima city	6/9/2011	37.8166	140.4149	9556±11	9392±11	0.042±0.002	1.017±0.002	0.339±0.019
S84	Fukushima city	6/9/2011	37.7614	140.4207	8330±10	8277±10	0.037±0.003	1.006±0.002	0.337±0.032
S85	Fukushima city	6/9/2011	37.7441	140.4322	11107±18	10982±18	0.049±0.003	1.011±0.002	0.340±0.021
S86	Fukushima city	6/9/2011	37.7518	140.4524	986±4	1001±4	0.005±0.001	0.985±0.005	0.400±0.092
S87	Fukushima city	6/10/2011	37.7591	140.3945	4150±11	4226±11	0.018±0.002	0.982±0.004	0.323±0.029
S88	Koriyama city	6/15/2011	37.3651	140.3314	11377±18	11643±18	0.055±0.005	0.977±0.002	0.361±0.031
S89	Asakanagamori Station	6/15/2011	37.3575	140.3686	6093±13	6251±13	0.029±0.001	0.975±0.003	0.355±0.007
S90	Koriyama city	6/15/2011	37.4164	140.3831	10471±17	10768±17	0.051±0.004	0.972±0.002	0.363±0.025
S91	Motomiya city	6/15/2011	37.4787	140.3732	8460±15	8360±15	0.037±0.001	1.012±0.003	0.333±0.009
S92	Koriyama city	6/16/2011	37.3980	140.3560	17232±18	17082±18	0.072±0.004	1.009±0.001	0.323±0.018
S94	Koriyama city	6/16/2011	37.3901	140.3766	16681±23	16431±23	0.077±0.004	1.015±0.002	0.357±0.021
L12	Minamisoma city	4/12/2011	37.5744	140.8822	205325±64	199163±65	0.881±0.020	1.031±0.000	0.337±0.008
L13	Namie town	4/12/2011	37.5419	140.8594	76162±44	73384±46	0.320±0.040	1.038±0.001	0.333±0.041
L14	Namie town	4/12/2011	37.5733	140.7931	190594±159	186103±166	0.817±0.150	1.024±0.001	0.335±0.062
L16	Namie town	4/13/2011	37.5538	140.7216	78169±125	75791±130	0.324±0.118	1.031±0.002	0.326±0.119
L19	Namie town	4/13/2011	37.5561	140.7398	186024±484	178921±501	0.794±0.134	1.040±0.004	0.338±0.057

Samples with an initial ID of “S” represent soil samples with activities in dry weight, while others represent plant samples with activities in wet weight.

^{134}Cs and ^{137}Cs activities were decay-corrected to March 11, 2011.

ND: Below the detection limit.

Table S2 | Evaluated amounts of released ^{137}Cs (PBq) due to the FDNPP accident, and inventory of ^{135}Cs (TBq) in the fuel of the reactor cores and the spent fuel pools (SFPs)

	**^{135}Cs inventory[a]**	**^{137}Cs released[b]**
Core-1	1.05	0.59
Core-2	1.14	14
Core-3	1.1	0.71
SFP-1	1.1	-
SFP-2	2.54	-
SFP-3	1.93	-
SFP-4	4.8	-

[a] Data from the Ministry of Economy, Trade and Industry [1]

[b] Data from the report of Nishihara *et al.*[2].

-No data.

Table S3 | The $^{134}Cs/^{137}Cs$ activity ratio in wet and dry precipitation depositions at eleven stations in Japan collected during the period from 9:00 on May 1 through 9:00 on June 1, 1986 (decay-corrected to March 11, 2011)

**Station**	**$^{134}Cs/^{137}Cs$ activity ratio ($\times 10^{-4}$)**
Wakkanai	2.02±0.33
Kushiro	2.08±0.34
Sapporo	2.05±0.36
Akita	2.13±0.34
Sendai	2.09±0.34
Tokyo	2.11±0.33
Wajima	2.12±0.34
Osaka	2.01±0.34
Yonago	2.03±0.35
Fukuoka	2.18±0.37
Ishigaki	2.61±0.60
Mean	2.13±0.17

Data from the report of Aoyama *et al.*[3].

Table S4 | The ^{135}Cs activities and $^{135}Cs/^{137}Cs$ atom ratios in soil samples before the FDNPP accident

ID	Contribution of global fallout	^{135}Cs (Bq kg^{-1})	$^{135}Cs/^{137}Cs$ atom ratio
S12	0.047±0.007	0.0178±0.0026	0.496±0.192
S13	0.047±0.008	0.0078±0.0014	1.616±0.471
S14	0.043±0.009	0.0076±0.0015	1.485±0.360
S15	0.042±0.008	0.0106±0.0019	0.731±0.297
S16	0.034±0.006	0.0060±0.0011	1.807±0.654
S19	0.025±0.017	0.0007±0.0005	2.720±2.879
S20	0.043±0.012	0.0025±0.0007	1.411±0.675
S23	0.038±0.012	0.0022±0.0008	1.911±1.039
S27	0.049±0.012	0.0026±0.0007	1.717±0.758
S29	0.043±0.016	0.0013±0.0005	2.044±1.246
S35	0.121±0.373	ND	ND
S36	0.105±0.066	ND	ND
S37	0.027±0.030	0.0002±0.0002	1.498±3.000
S39	ND	ND	ND
S40	0.526±0.044	ND	ND
S42	0.040±0.011	0.0036±0.0010	2.058±0.924
S44	ND	ND	ND
S45	0.049±0.010	0.0048±0.0010	1.302±0.425
S46	0.063±0.007	0.0212±0.0024	0.533±0.159
S47	0.043±0.008	0.0085±0.0016	1.462±0.430
S48	0.025±0.009	0.0088±0.0032	0.949±0.475
S49	0.050±0.007	0.0153±0.0023	0.606±0.182
S54	0.003±0.008	0.0017±0.0038	4.021±9.638
S55	0.025±0.021	0.0003±0.0003	2.339±2.580
S56	0.054±0.028	0.0004±0.0002	1.375±1.161
S57	0.021±0.014	0.0008±0.0005	3.386±2.455
S58	0.042±0.015	0.0014±0.0005	2.387±1.240
S59	0.026±0.012	0.0017±0.0008	2.707±1.555
S60	0.046±0.033	0.0002±0.0002	0.651±1.271

S61	0.069±0.016	0.0013±0.0003	0.462±0.634
S62	0.044±0.006	0.0041±0.0006	0.641±0.289
S63	0.033±0.006	0.0086±0.0017	0.827±0.461
S64	0.039±0.008	0.0002±0.0001	1.847±3.295
S65	0.048±0.009	0.0005±0.0002	0.779±1.645
S66	0.048±0.006	0.0034±0.0005	0.458±0.284
S67	0.039±0.006	0.0048±0.0008	0.374±0.224
S68	0.043±0.006	0.0018±0.0003	0.253±0.440
S69	0.049±0.006	0.0041±0.0005	0.150±0.341
S70	0.060±0.006	0.0028±0.0004	0.480±0.630
S71	0.065±0.006	0.0069±0.0007	0.378±0.158
S72	0.061±0.007	0.0016±0.0003	0.748±0.839
S73	0.010±0.006	0.0006±0.0004	2.995±2.973
S74	0.052±0.006	0.0054±0.0008	0.430±0.520
S75	0.044±0.007	0.0017±0.0003	0.870±0.648
S76	0.013±0.006	0.0007±0.0004	0.127±1.750
S77	0.021±0.006	0.0024±0.0007	0.028±0.907
S78	0.036±0.008	0.0003±0.0001	
S79	0.040±0.006	0.0033±0.0005	0.397±0.361
S80	0.023±0.006	0.0016±0.0005	0.634±0.598
S81	0.038±0.006	0.0019±0.0003	0.362±0.365
S82	0.012±0.006	0.0007±0.0004	1.069±1.168
S83	0.015±0.006	0.0006±0.0003	0.667±1.355
S84	0.026±0.006	0.0009±0.0002	0.468±1.275
S85	0.021±0.006	0.0010±0.0003	0.638±1.049
S86	0.046±0.008	0.0002±0.0001	1.766±2.021
S87	0.049±0.007	0.0009±0.0001	0.110±0.610
S88	0.054±0.006	0.0030±0.0004	0.844±0.594
S89	0.056±0.007	0.0016±0.0002	0.706±0.174
S90	0.058±0.006	0.0030±0.0004	0.831±0.454
S91	0.020±0.007	0.0007±0.0002	0.309±0.511
S92	0.023±0.006	0.0017±0.0005	
S94	0.017±0.006	0.0013±0.0005	1.700±1.414

ND: Below the detection limit.

Contribution of global fallout was calculated according to equation (1); it should be noted that the uncertainties of some samples with extremely low radiocesium activities were therefore somewhat higher due to error propagation. The sum of contributions from global fallout Cs and the FDNPP accident derived fallout was considered as 1.

Table S5 | Optimized analytical parameters of ICP-MS/MS

**Plasma**	
RF power	1550 W
RF matching	1.70 V
Sampling position	10.0 mm
Carrier gas	1.15 L min^{-1}
Nebulizer pump	0.2 rps
Spray chamber temperature	2^{0}C
Makeup gas flow rate	0.10 L min^{-1}
**Lens**	
Extraction lens 1	-2.0 V
Extraction lens 2	-190.0 V
Omega bias	-105 V
Omega lens	10.7V
Q1 entrance	3 V
Q1 exit	1 V
Cell focus	5.0 V
Cell entrance	-50 V
Cell exit	-60 V
Deflection	1.8 V
Plate bias	-60 V
**4th cell gas line**	
N_2O flow rate	0.54 mL min^{-1}
**Integration time**	
m/z = 95, 118, 121, 133, and 138	1 s
m/z = 134, 135, and 137	5 s

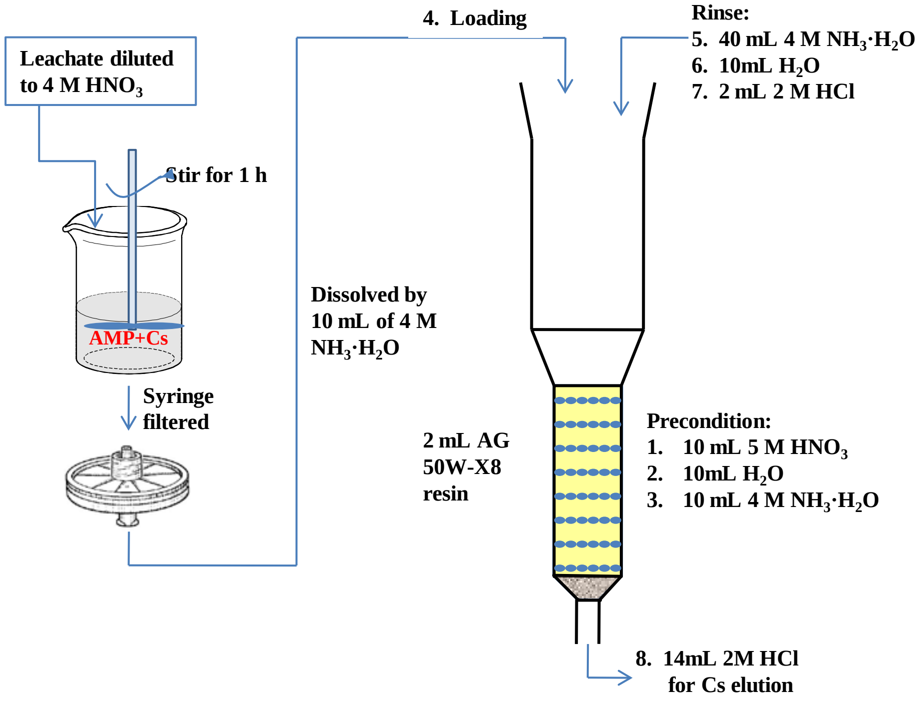

Figure S1 | Summary of chemical separation for ^{135}Cs and ^{137}Cs, including AMP selective adsorption and cation exchange chromatography [4].

References

1. Ministry of Economy, Trade and Industry. Data on the amount of released radioactive materials, (2011) Available at: http://warp.ndl.go.jp/info:ndljp/pid/6086248/www.meti.go.jp/press/2011/10/20111020001/20111020001.pdf (Accessd: 3/8/2016).
2. Nishihara, K., Iwamoto, H. & Suyama, K. Estimation of fuel compositions in Fukushima-Daiichi Nuclear Power Plant. JAEA-Data/Code 2012-018, 65–117 (2012).
3. Aoyama, M., Hirose. K. & Sugiyama, Y. Deposition of gamma-emitting nuclides in Japan after the reactor-IV accident at Chernobyl. *J. Radioanal. Nucl. Chem.* **116**, 291–306 (1987).
4. Yang, G. S., Tazoe, H. & Yamada, M. Rapid determination of ^{135}Cs and precise ^{135}Cs/^{137}Cs atomic ratio in environmental samples by single-column chromatography coupled to triple-quadrupole inductively coupled plasma-mass spectrometry. *Anal. Chim. Acta* **908**, 177−184 (2016).
